# Supplementary material for: Chromothripsis during telomere crisis is independent of NHEJ, and consistent with a replicative origin
Source: Genome Res. 2019 May;29(5):737–49. doi: 10.1101/gr.240705.118 (PMC6499312; doi:10.1101/gr.240705.118)
Supplement: Supplemental Material [file supp_gr.240705.118_Supplemental_file_1.zip › contigs/annotated_contigs/DB57/contig.2.DB57_length_469_mean_cov_4.63113006397.docx]

**DB57_length_469_mean_cov_4.63113006397**

```
TTGGATGGAACCACCAAACTATCTAGTTTGGGTTCTTCTTGACATCTATGTCTCCAGGGCTTCGCTTTCTTCTTTAAGTAGTTACTATC
 >chr11:18868116-18868409 + E=7e-165
CTTGCCACATTTAATGAATATGTTTAAGCTTCCAGAAATCCTTCAAGGTAGAAGGCAGGGTGGTTAAAACCTGACAGTTTTCTGCTCAT

GTTTTTGGAACGTGACACCTGATGGTTGAGGTTGCTGAAGATGCACTCCAAAGATGCATGGGGGTAGAATAAAGGGGAGAAGATGGTGG

TCCTTTTGGAATTTGGGTTGTGCCAA|ATTCA|GAAAAATCTGAATGCTGTGACACTACCAAAGGATCACACTAGATCTCTAGCAATGG
                                >chr11:18875335-18875506 + E=2e-91
TCTCTAACCAAAATGGAAACTCAGAAATGACAGATAAAGAATTCAAAGCATTGATCACAGGGAAGCTCAGTGAGATCCAAGACAAGGTT

GAAAATCAACACAAAGATACTTCTAA
```
